# Body mass index combined with waist circumference can predict moderate chronic kidney disease: A retrospective study

**DOI:** 10.1097/MD.0000000000025017

**Published:** 2021-03-26

**Authors:** Hong Cai, Yaping Zhan, Jiayue Lu, Minxia Zhu, Shang Liu, Jiuhong Mei, Pu Zhang, Ronghui Liu, Zhaohui Ni, Weiming Zhang, Gaojie Xu

**Affiliations:** a Department of Nephrology, Renji Hospital, School of Medicine, Shanghai Jiao Tong University; b Department of General Medicine, Shanghai Pudong New Area Yingbo Community Health Service Center, Shanghai, China.

**Keywords:** body-mass index, chronic kidney disease, waist circumference, waist-to-hip ratio

## Abstract

Overweight and obesity may be associated with poor clinical outcome, including chronic kidney disease (CKD). However, whether body mass index (BMI), waist-to-hip ratio (WHR), and waist circumference (WC) are related to CKD is yet to be elucidated.

A total of 7593 adults were divided into 4 groups based on the estimated glomerular filtration rate (eGFR) quartile. The eGFR was calculated with the CKD Epidemiology Collaboration. Multiple linear regression analyzed the association between eGFR and WHR, BMI, and WC. Logistic regression analysis determined whether the CKD patients were associated with WHR, BMI, and WC after adjusting for other variables.

The mean age of the cohort was 72.34 ± 7.30 years. Multiple linear regression analysis showed that WC (*P* = .006) was associated with eGFR, although adjusted by lifestyle factor and biochemical indicators. The individuals in the underweight, overweight, and obese groups had significantly lower eGFR value than those in the healthy weight group in moderate CKD. The eGFR in the overweight group with WHR ≤0.894 was higher than in the healthy weight group with WHR >0.894 group (*P* = .036). Overweight with WHR ≤0.894 group had a longer WC with a pronounced increase in the hip circumference. Logistic regression analysis showed that the WC (OR = 1.362, *P* < .001) and BMI (OR = 1.227, *P* = .031) were independent risk factors for moderate CKD patients. Each standard deviation (SD) of high BMI and WC level was associated with 23.0% and 17.3% higher odds of moderate CKD (OR = 1.230, *P* = .017 and OR = 1.173, *P* = .021, respectively).

WC is an independent risk factor for eGFR. Combined BMI and WC are important factors that would predict moderate CKD patients.

## 1 Introduction

Chronic kidney disease (CKD) is increasing in prevalence and incidence and is recognized as a public health priority worldwide.^[1,2]^ Overweight and obesity are closely related to CKD.^[1]^ The links between them are numerous and complex. This complexity maybe explained by inflammation, increased oxidative, insulin resistance, hypertension and dyslipidemia.^[3]^

Body mass index (BMI) is a critical and convenient indicator for assessing whether a subject is overweight or obese. High BMI contributed to an estimated 4 million deaths globally in 2015.^[4]^ Several studies have shown a U-shaped association between BMI and minimum mortality in the healthy weight (20–25 kg/m^2^) range.^[5–7]^ The BMI also had a J-shaped association with the overall mortality and the specific causes of death.^[8]^ Recent studies have shown that BMI may be a predictor of kidney disease. BMI ≥30 kg/m^2^ was associated with the rapid loss of kidney function in a patient with eGFR ≥60 ml/minutes/1.73 m^2^.^[9]^ However, other studies demonstrated that waist-to-hip ratio (WHR), but not BMI, is associated with incident chronic kidney disease (CKD). The evaluation of the risk of CKD utilized WHR rather than BMI as an anthropomorphic measure of obesity^[10]^ as it is an indirect measure of visceral fat, whereas BMI does not distinguish between visceral and non-visceral fat. An increased WHR indicates decreased gluteal muscle mass or increased visceral fat; therefore, the contribution of visceral fat and gluteal muscle to WHR was relevant. Studies in Waist circumference (WC) which also can measure visceral abdominal fat show that WC is a strong predictor of adverse events and has relationship with adverse clinical outcomes in CKD.^[11]^ In general population and CKD patients, WC has well correlated with visceral adipose tissue. However, the majority of the patients with CKD were stage CKD1–2. The best index in predicting moderate CKD (eGFR <60 ml/minutes/1.73 m^2^) patients is unknown. Thus, in this study, we focus on the moderate CKD patients and measured the WC, hip circumference, BMI, and calculated the WHR to find how we can better predict the risk factors for the development of kidney disease.

## 2 Methods

### 2.1 Participants

**Figure 1:**
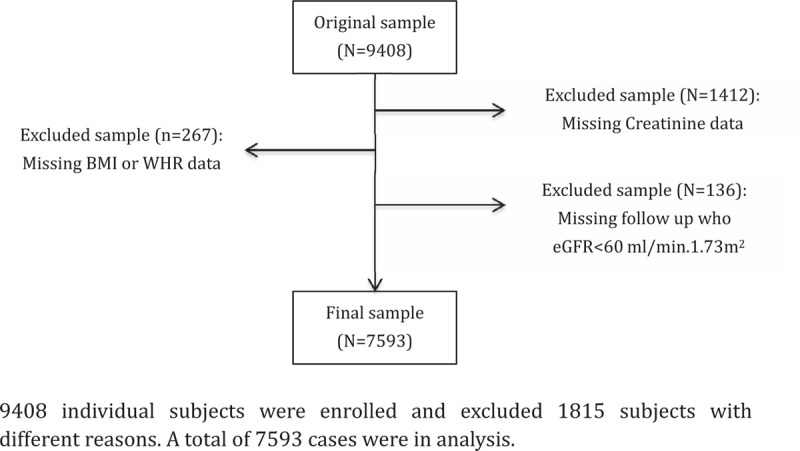
Flowchart describing sample selection 9408 individual subjects were enrolled and excluded 1815 subjects with different reasons. A total of 7593 cases were in analysis.

The data from individual subjects were pooled from Shanghai Pudong new area Yingbo community healthy examination from 2015 to 2018 and the study was approved by the Ethics Committee on human of Shanghai Pudong new area Yingbo community health service. All the patients provided written informed consent to participate in this study. The methods were carried out in accordance with the approved guidelines. A total of 9408 subjects, aged 12 to 93 years, were recruited in this study. Subject with a low estimated glomerular filtration rate (eGFR) (<60 ml/minutes/1.73 m^2^) at the first examination were followed up after 3 months. CKD was defined as eGFR <60 ml/minutes/1.73 m^2^ for >3 months. According to CKD stage, eGFR <60 ml/minutes/1.73 m^2^ was defined as moderate CKD. Individuals lacking data on creatinine, sex, and age were excluded from the pool (n = 1412). Also, 267 cases were excluded due to the missed BMI or WHR data. People who missed the 3-month follow-up were also excluded from the data (n = 136). Finally, a total of 7593 cases were included in the current analysis (Fig. 1).

### 2.2 Variables

WHR was calculated as WC divided by hip circumference; both were measured according to the recommendations of the World Health Organization (WHO). The subject was told to stand relaxed with arms at the sides, feet positioned close together, and weight distributed across the feet. WC was measured between the lowest rib and the superior border of the iliac crest. Hip circumference was measured at the widest portion of the buttocks. The BMI was calculated as weight in kilograms (kg) divided by squared length in meters (m^2^). Obese, overweight, healthy weight, and underweight were defined as BMI ≥30.0, 25.0 to 29.9, 18.5 to 24.9, and <18.5 kg/m^2^, respectively.^[12]^ Estimated GFR was calculated using the CKD Epidemiology Collaboration creatinine 2009 equation.^[13]^

The baseline variables included demographics (age, sex), lifestyle characteristics (smoking, alcohol use), medical history (diabetes, hypertension), and examination findings (systolic and diastolic blood pressure), as well as laboratory variables (hemoglobin, albumin (Alb), creatinine, total cholesterol, high density lipoprotein (HDL), low density lipoprotein (LDL), and triglyceride). Cigarette smoking and alcohol consumption was dichotomized as current users and non-users. Diabetes was defined as a history of previous diagnosis and use of an oral hypoglycemic agent or insulin. Hypertension was defined as have a history of diagnosis, systolic blood pressure ≥140 mm Hg, diastolic ≥90 mm Hg, or use of antihypertensive medications.

### 2.3 Statistical analysis

Continuous variables were described as mean ± SD or medians with interquartile range (IQR), depending on their distributions, while categorical variables were expressed as numbers (percentages). The differences in demographic data and clinical variables across the eGFR quartiles were analyzed using the independent-samples *t*-test for continuous variables and the Chi-Squared test or Fisher exact test for categorical variables. Multiple linear regression analysis was adjusted for potential confounders to assess the association between eGFR and WHR, BMI, and WC. A binary logistic regression analysis was performed to determine the association of moderate CKD with BMI, WC, and WHT, adjusting for demographic and clinical variables. All the variables were set to binary dependent variable. To compare the effects of WHR, BMI, and WC, risks were presented also per standard deviation (SD) increase in the independent variable. *P* < .05 was considered statistically significant. SPSS 20.0 was used to compute the statistical analyses.

## 3 Results

### 3.1 Baseline characteristics

The mean of the cohort was 72.34 ± 7.30 years. The mean BMI, WHR, and WC were 23.91 ± 5.04 kg/m^2^, 0.90 ± 0.15, and 85.50 ± 10.71 cm, respectively. The mean baseline serum creatinine was 74.30 ± 23.13 mmol/L, and mean eGFR was 81.54 ± 17.58 ml/minutes/1.73 m^2^. As anticipated, individuals with low eGFR were likely to have a high prevalence of smoking, alcohol use, and high systolic blood pressure (SBP). The low level of hemoglobin (Hb), Alb, and HDL are associated with low eGFR (Table 1).

**Table 1:** In whole cohort, Baseline characteristics of community-living individuals and laboratory data by quartiles of eGFR quartiles.

		eGFR quartiles (ml/min 1.73 m^2^)			
	All (n = 7593)	I≤70.76 (n = 1899)	70.76<II≤83.25 (n = 1898)	83.25<III≤92.68 (n = 1898)	IV>92.68 (n = 1898)
Demographics					
Male, n (%)	3427 (45.1)	822 (43.3)	879 (46.3)	633 (33.4)^∗∗^	1093 (57.6)^∗∗^
Age, yr	72.34 ± 7.30	76.34 ± 7.80	73.13 ± 6.90^∗∗^	71.49 ± 6.06^∗∗^	68.39 ± 5.95^∗∗^
Smoker, n (%)	1460 (19.2)	511 (26.9)	374 (19.7)^∗∗^	275 (14.5)^∗∗^	300 (15.8)^∗∗^
Hypertension, n (%)	2504 (33.0)	637 (33.5)	584 (30.3)^∗^	620 (32.7)	663 (34.9)
Alcohol Use, n (%)	2224 (29.3)	651 (34.3)	640 (33.7)	538 (28.3)^∗∗^	395 (20.8)^∗∗^
Diabetes, n (%)	672 (8.85)	162 (8.53)	175 (9.23)	168 (8.85)	167 (8.80)
Physical Findings					
SBP, mm Hg	141.43 ± 21.18	143.39 ± 21.67	141.73 ± 20.22^∗^	140.91 ± 20.72^∗∗^	139.62 ± 21.90^∗∗^
DBP, mm Hg	79.42 ± 14.80	78.28 ± 11.96	79.18 ± 11.81	79.70 ± 15.06^∗∗^	80.58 ± 19.24^∗∗^
BMI, kg/m^2^	23.91 ± 5.04	23.97 ± 0.91	23.82 ± 0.90	23.97 ± 0.89	23.88 ± 0.90
WHR	0.89 ± 0.15	0.91 ± 0.16	0.90 ± 0.11^∗∗^	0.89 ± 0.08^∗∗^	0.90 ± 0.21^∗^
WHR (Male)	0.91 ± 0.10	0.92 ± 0.07	0.91 ± 0.14^∗^	0.91 ± 0.08^∗^	0.91 ± 0.08^∗∗^
WHR (Female)	0.89 ± 0.18	0.90 ± 0.21	0.88 ± 0.77^∗^	0.88 ± 0.08^∗∗^	0.89 ± 0.08^∗^
WC (cm)	85.50 ± 10.71	86.49 ± 9.88	85.49 ± 12.29^∗∗^	84.71 ± 10.42^∗∗^	85.28 ± 10.00^∗∗^
WC (cm) (Male)	87.49 ± 11.10	88.89 ± 9.48	87.79 ± 14.84^∗^	87.17 ± 9.15^∗∗^	86.38 ± 9.5^∗∗^
WC (cm) (Female)	83.88 ± 10.10	84.66 ± 9.80	83.52 ± 9.13^∗^	83.58 ± 10.80^∗^	83.79 ± 10.47^∗^
Baseline Laboratory Results					
Scr, mmol/l	74.30 ± 23.13	96.44 ± 31.99	75.98 ± 10.46^∗∗^	64.73 ± 10.31^∗∗^	60.02 ± 10.58^∗∗^
eGFR, ml/min 1.73 m^2^	81.54 ± 17.58	58.92 ± 10.33	77.30 ± 3.63^∗∗^	88.20 ± 2.75^∗∗^	101.74 ± 11.26^∗∗^
Hb, g/l	136.18 ± 15.00	132.55 ± 17.27	136.63 ± 14.70^∗∗^	136.42 ± 13.41^∗∗^	138.96 ± 13.91^∗∗^
Alb, g/l	46.25 ± 3.30	45.97 ± 3.46	46.27 ± 3.26	46.26 ± 3.12	46.51 ± 3.34^∗∗^
TC, mmol/l	4.71 ± 2.00	4.69 ± 1.58	4.68 ± 1.09	4.82 ± 2.02	4.62 ± 2.22
TG, mmol/l	1.73 ± 1.64	1.77 ± 1.23	1.71 ± 1.21	1.81 ± 1.57	1.63 ± 1.14
LDL, mmol/l	2.52 ± 0.86	2.52 ± 0.88	2.54 ± 0.84	2.55 ± 0.87	2.47 ± 0.85
HDL, mmol/l	1.35 ± 0.34	1.32 ± 0.33	1.36 ± 0.35^∗∗^	1.38 ± 0.34^∗∗^	1.36 ± 0.33^∗^

^∗^Compared with group I *P* < .05; ^∗∗^ Compared with group I *P* < .001 SBP = systolec blood pressure, DBP = diastolec blood pressure, BMI = body mass index, WHR = waist hip ratio, WC = Waist Circumference, Hb = haemoglobin, Alb = albumin, Scr = serum createnine, eGFR = estimated glomerular filtration rate, TC = total cholesterol, TG = triglyceride, LDL = low density lipoprotein cholesterol, HDL = high density lipoprotein.

### 3.2 Multiple linear regression analysis with eGFR and WHR, WC, BMI

All the individual subjects were enrolled to identify the association between WC, BMI, WHR with eGFR, a multiple linear regression analysis was performed (Table 2). Age (β = −1.039, 95% confidence interval (CI): −1.117 to −0.962, *P* < .001), Hb (β = 0.112, 95% CI: 0.074–0.150, *P* < .001), HDL (β = 1.931, 95% CI: 0.240–5.115, *P* = .025), WC (β = −0.066, 95% CI: −0.113 to −0.019, *P* = .006) were associated with the level of eGFR after adjusting for lifestyle factor and biochemical indicators(Table 2).

**Table 2:** Multiple linear regression analysis of risk factors for serum estimated GFR.

	Univariate analysis		Multi variate analysis	
	β	*P*	β	*P*
Age	−0.957 (−1.007, −0.907)	<.001	−1.039 (−1.117, −0.962)	<.001
SBP	−0.05 (−0.07, −0.031)	<.001		
DBP	0.07 (0.042, 0.099)	<.001		
Hb	0.234 (0.204, 0.265)	<.001	0.112 (0.074, 0.150)	<.001
Alb	0.380 (0.214, 0.545)	<.001		
TC	0.144 (−0.071, 0.359)	.088		
TG	−0.229 (−0.491, 0.034)	.088		
HDL	1.776 (0.504, 3.048)	0.006	1.931 (0.240, 5.115)	.025
LDL	−0.162 (−0.663, 0.339)	.527		
BMI	0.002 (−0.078, 0.082)	.964		
WHR	−3.436 (−6.147, −0.726)	.013		
WC	−0.083 (−0.121, −0.045)	<.001	−0.066 (−0.113, −0.019)	.006

Statistical model used multiple linear regression analysis for measure the risk of serum estimated GFR adjusting for demographic data and clinical data. SBP = systolec blood pressure, DBP = diastolec blood pressure, Hb = haemoglobin, Alb = albumin, TC = total cholesterol, TG = triglyceride, LDL = low density lipoprotein cholesterol, HDL = high density lipoprotein, BMI = body mass index, WHR = waist-to-hip Ratio, WC = Waist Circumference.

### 3.3 Association of moderate CKD with BMI, WC and WHR

Univariate analysis showed that moderate CKD patients were correlated with WC, WHR, and BMI. Logistic regression analysis showed that WC level (hazard ratio (HR) = 1.362, *P* = .001), BMI level (HR = 1.227, *P* = .031), smoker (HR = 1.551, *P* = .002), male gender (HR = 1.416, *P* = .015), and the level of Hb (HR = 0.437, *P* < .001) and Alb (HR = 0.798, *P* = .044) were independent risk factors for the moderate CKD patients after adjusting for demographics and biochemical indexes (Table 3).

**Table 3:** Analysis of risk factor for moderate CKD.

	Unadjusted			Adjusted 1			Adjusted 2		
	OR	95% CI	*P* value	OR	95% CI	*P*	OR	95% CI	*P* value
WC	1.270	1.084–1.489	.003	0.894	0.652–1.213	.091	1.362	1.215–1.526	<.001
WHR	1.244	1.161–1.334	<.001	1.466	1.193–1.801	<.001	1.212	0.825–1.691	.661
BMI	1.246	1.118–1.390	<.001	1.254	1.083–1.452	.002	1.227	1.129–1.628	.031
Smoker				1.724	1.082–1.991	.005	1.551	1.199–2.591	.002
Alcohol				1.141	0.617–4.812	.493	1.112	0.401–3.989	.626
Male				1.225	0.694–3.811	.628	1.416	1.071–1.873	.015
Diabetes				1.228	0.724–5.221	.361	1.374	0.628–4.259	.249
Age							1.488	0.523–6.968	.321
SBP							1.201	0.824–1.879	.320
DBP							0.662	0.364–1.098	.221
Hb							0.437	0.335–0.571	<.001
Alb							0.798	0.532–0.922	.044
TC							0.724	0.445–1.187	.375
TG							1.233	0.784–1.663	.275
LDL							1.103	0.751–1.557	.555
HDL							0.839	0.647–1.429	.093

Statistical model used logistic regression analysis for measure the risk of moderate CKD patients adjusting for demographic data and clinical data.(Set WC ≤85.5 cm = 0, WC >85.5 cm = 1;WHR ≤0.894 = 0, WHR >0.894 = 1, BMI ≤24.90 = 0, BMI >24.9 = 1; Smoker = 1, no smoker = 0; Alochol = 1, no Alochol = 0; Male = 1, Female = 0, Diabetes = 1non-Diabetes = 0, Age ≤72.34 (y) = 0, Age >72.34(y) = 1, SBP ≤141.43 mm Hg = 0, SBP >141.43 mm Hg = 1, DBP ≤79.42 mm Hg = 0, DBP >79.42 mm Hg=1, Hb ≤136.18 (g/l) = 0, Hb >136.18 (g/l) = 1, Alb ≤46.25 g/l = 0, Alb >46.25 g/l = 1, TC ≤4.71 mmol/l = 0, TC >4.71 mmol/l=1, TG ≤1.73 mmol/l = 0, TG >1.73 mmol/l=1, LDL ≤2.52 mmol/l = 0, LDL >2.52 mmol/l=1, HDL ≤1.35 mmol/l=0, HDL >1.35 mmol/l = 1) WHR = Waist-to-hip Ratio, BMI = body mass index, SBP = systolec blood pressure, DBP = diastolec blood pressure, Hb = haemoglobin, Alb = albumin, TC = total cholesterol, TG = triglyceride = LDL = low density lipoprotein cholesterol, HDL = high density lipoprotein.

### 3.4 Correlation between different levels of BMI, WHR, and the value of eGFR in moderate CKD

Incidence of moderate CKD and lower eGFR value were higher in both underweight and overweight/obese people than in healthy weight people. The healthy weight with WHR ≤0.894 group had the highest value of eGFR as compared to the other groups. The value of eGFR in the overweight with WHR ≤0.894 group was higher than in the healthy weight with WHR >0.894 group (*P* = .036). Although the obese with WHR ≤0.894 group (n = 23) had a higher value of eGFR, it was not statistically significant as compared to the healthy weight with WHR >0.894 group. In addition, compared to the healthy weight with WHR >0.894, overweight with WHR ≤0.894 group had a longer WC with a pronounced increase in the hip circumference (Table 4).

**Table 4:** The relationship between eGFR and BMI, Waist circumference, Hip circumference in people with moderate CKD.

		Underweight (<18.5 kg/m^2^)	Healthy weight (18.5–24.9 kg/m^2^)	Overweight (25.0–29.9 kg/m^2^)	Obese (⩾30.0 kg/m^2^)
Moderate CKD (n, %)	774 (10.2)	45 (12.1)^&^	373 (8.6)	288 (11.5)^&^^&^	68 (17.2)^&^^&^
eGFR (ml/min 1.73 m^2^)	All	48.92 ± 10.38	51.28 ± 9.43	49.82 ± 9.87	47.66 ± 9.37
	WHR ≤0.894	53.63 ± 9.62	52.73 ± 7.84^#^	51.22 ± 9.86^#^	49.76 ± 9.39
	WHR >0.894	47.10 ± 12.04	48.85 ± 9.76^∗^	48.16 ± 9.81^∗^	44.63 ± 13.75
Waist Circumference (cm)	All	72.34 ± 13.62^&^^&^	81.59 ± 6.97	93.09 ± 7.89^&&^	98.42 ± 10.23^&&^
	WHR ≤0.894	67.81 ± 6.38^∗∗##^	78.53 ± 6.57^##^	86.33 ± 7.76^∗∗#^	77.50 ± 10.61
	WHR >0.894	78.14 ± 19.70	82.58 ± 6.81^∗∗^	93.72 ± 7.65^∗∗##^	99.09 ± 9.57^∗∗##^
Hip Circumference (cm)	All	81.83 ± 20.95^&^^&^	91.79 ± 6.11	99.53 ± 6.30^&^^&^	103.92 ± 7.85^&^^&^
	WHR ≤0.894	84.63 ± 4.36^∗∗##^	93.20 ± 5.43^#^	100.13 ± 6.55^∗∗##^	90.00 ± 7.07
	WHR >0.894	87.93 ± 17.80^∗^	91.33 ± 6.25^∗^	99.48 ± 6.29^∗∗##^	104.36 ± 7.51^∗∗##^

^∗^compared with group of Healthy weight and WHR ≤0.894, *P* < .05, ^∗∗^ compared with group of Healthy weight and WHR≤0.894, *P* < .01; ^#^ compared with group of Healthy weight and WHR >0.894, *P* < .05, ^##^ compared with group of Healthy weight and WHR >0.894, *P* < .01 ^&^ compared with group of Healthy weight (All), *P* < .05, ^&^^&^ compared with group of Healthy weight (All), *P* < .01;

Each SD of high BMI and WC level was associated with 23.0% and 17.3% higher odds of moderate CKD in a model adjusted for lifestyle factors and biochemical indicators (odds ratio (OR) = 1.230, *P* = .017 and OR = 1.173, *P* = .021, respectively) (Table 5).

**Table 5:** Association between moderate CKD and WHR, BMI, WC.

	WHR per SD (0.147) greater		BMI per SD (5.07 kg/m^2^)greater		WC per SD (10.763) greater	
Model	OR	*P*	OR	*P*	OR	*P*
Unadjusted	1.056 (1.002, 1.113)	.043	1.061 (0.983, 1.146)	.129	1.200 (1.107, 1.301)	<.001
Life Adjusted^∗^	1.080 (0.998, 1.170)	.058	1.115 (1.007, 1.234)	.036	1.138 (1.043, 1.242)	.004
Full Adjusted^∗∗^	1.011 (0.923, 1.107)	.841	1.230 (1.037, 1.458)	.017	1.173 (1.024, 1.344)	.021

∗ Adjusted for age, sex, smoking, Alcohol Use,

∗∗ Adjusted for lifestyle model plus BMI, Hb, Alb, TC, TG, HDL, LDL, SBP, DBP. Multivariate logistic regression analysis of moderate CKD patients. After adjusting lifestyle and clinical data, each SD of high BMI and WC level but not WHR was higher risk of moderate CKD.

## 4 Discussion

Due to the rising prevalence of CKD, the prevention, treatment, and detection of the disease in the early stage is under the focus of the doctor. Overweight and obesity are closely related to CKD according to the epidemiological studies. Overweight, obesity, diabetes, cardiovascular disease, and some tumors have been listed as the focus of global public health. In the present study, irrespective of the gender and in the general population, the WHR of patients with eGFR <70 ml/minutes/1.73 m^2^ was significantly higher than that of patients with higher eGFR. Compared to BMI, WHR exhibited a correlation with the progress in renal function. In creatinine-based models, WHR increased the risk of CKD by 22%, albeit no significant correlation was established between BMI and CKD. Therefore, the researchers suggested using WHR instead of BMI as a predictor of CKD.^[10]^

This phenomenon might be attributed to the inability of BMI to distinguish among peripheral fat, visceral fat, and muscle tissue.^[14,15]^ The WHR can indirectly reflect visceral obesity, which is an independent risk factor for cardiovascular events in non-dialysis patients. Visceral obesity is also an independent risk factor for cardiovascular disease in the normal population. However, multivariate linear regression analysis found that WC, not WHR, was an independent risk factor for eGFR after correcting the general conditions and biochemical indicators. Although WHR can indirectly respond to visceral obesity, it cannot distinguish whether the increase in WHR is due to decreased gluteal muscle mass or increased visceral fat. In the general population, the most widely used measure of central fat distribution is waist circumference, which is easier to determine than WHR and provides equivalent prognostic information.^[16]^ Interestingly, unlike WHR, WC is an established criterion for the diagnosis of metabolic syndrome. One study suggested that WC and visceral obesity are closely related in CKD patients, with a correlation coefficient of 0.75 for men and 0.81 for women.^[17]^ In a study consisting of 4222 participants, the increase in BMI and WHR was closely related to the incidence of CKD, after adjusting for smoking, drinking, age, and sex. However, the majority of the patients with CKD in this study were stage CKD1-2, while only a small number of patients had eGFR <60 ml/minutes/1.73 m^2^.^[18]^ In this study, we focus on the moderate CKD patients and the moderate CKD patients with WC and BMI as independent risk factors for predicting the occurrence of CKD; even if the general situation and biochemical indicators were corrected, the OR values were 1.362 and 1.227, respectively. Therefore, WHR may not be an optimal indicator for moderate CKD patients.

BMI is recommended by the WHO to assess whether a patient is overweight or obese. BMI (at >25 and <30 kg/m^2^, and >30 kg/m^2^ was defined as overweight and obesity, respectively.^[14]^ The correlation between BMI and the all-cause mortality in healthy population showed an anti-J shape. Compared to normal BMI (>18.5, <25 kg/m^2^), BMI <18.5 kg/m^2^ and >30 kg/m^2^ significantly increased the risk of all-cause mortality.^[12]^ However, high BMI might also affect the progress of the renal function. Xie et al showed that the risk of renal function progress increased by 82% in hypertensive patients with BMI >28.0 kg/m^2^ with normal renal function. Increased BMI was an independent risk factor for renal function progression.^[19]^ Intriguingly, the effect of BMI on renal function may also be related to age. The higher the BMI, the more severe the loss of renal function and the higher the mortality in patients >40-years-old. BMI and renal function did not seem to correlate in younger patients (<40-years-old). However, in the current study, BMI was not significantly correlated with eGFR, but BMI was found to be an independent risk factor for moderate CKD. For every SD add to BMI, the risk of CKD increased by 23.0%. This correlation persisted even if habits and biochemical indicators were corrected. In a recent study of 3,376,187 subjects, BMI (>30 kg/m^2^) was found to be closely related to the rapid loss of renal function in patients with eGFR (>60 ml/minutes/1.73 m^2^).^[20]^ In the current study, eGFR of people with moderate CKD showed an inverted “J” pattern. BMI in the 18.5 to 24.9 kg/m^2^ group exhibited the maximal eGFR level. With the gradual increase and decrease of BMI, the level of eGFR was gradually decreased.

Since WC and BMI are related indicators of metabolic syndrome and obesity, this study evaluated the correlation between obesity and CKD. Although there are reports that WHR is associated with the occurrence of CKD, in this study, WC is an independent risk factor affecting eGFR and predicting CKD after correcting the general condition and biochemical indicators of the patients. WC is strongly correlated with visceral fat and an independent risk factor for cardiovascular events in CKD patients.^[14]^ High WHR cannot differentiate between lower gluteal muscle due to malnutrition or visceral obesity. In the present study, the level of eGFR of overweight and WHR <0.894 groups was significantly higher than that of healthy weight and WHR >0.894 groups. Although the WC in the former group was slightly larger than that in the latter group, and the hip circumference of the former was significantly larger than that of the latter group. This phenomenon indicated that the overweight in the overweight and WHR <0.894 groups was caused by the increase in hip circumference, and not by the increase in WC. The WHR cannot distinguish the correlation between visceral obesity and gluteal muscles, and hence, WC has more predictive value. Multiple linear regression analysis also supported that WC is an independent risk factor for predicting the eGFR level.

In the current study, high BMI is an independent risk factor for predicting CKD, which is caused by the increase in renal sinus fat,^[21]^ glomerulomegaly,^[22]^ and glomerular hypertension.^[23]^ Overweight and obese patients increase the risk of hypertension and diabetes mellitus, which leads the kidney to have high perfusion and filtration. Hyperfiltration in patients with high BMI often occurs at an early age in the case of prehypertension and prediabetes.^[24]^ The structural and physiological changes in nephrons result from obesity, which might occur after prolonged exposure, followed by irreversible pathological change.^[25]^ Therefore, long-time and continuous control of ideal BMI is an effective means to protect the kidney.

Nevertheless, the present study has several limitations. First, the definition of CKD in the study was limited to patients with eGFR <60 ml/minutes/1.73 m^2^. Due to the lack of data on urine protein, the prevalence of CKD might be underestimated. Second, this study mainly focused on patients with eGFR <60 ml/minutes/1.73 m^2^. The lack of data on patients with eGFR>60 ml/minutes/1.73 m^2^ might not reflect the characteristics of CKD patients. Third, there was no long-term follow-up in this study. Whether BMI and WC predicted the prognosis of CKD patients and CVD complications is yet unknown. Finally, the subjects in this study were urban residents, which could not represent the characteristics of the vast rural population. Thus, we need to continue to follow up patients to observe if BMI and WC can predict the occurrence of cardiovascular events and the progression of kidney disease.

In conclusion, the association between BMI and eGFR in this cohort of patients with CKD is anti-J shaped. WC is an independent risk factor for eGFR. BMI and WC are important factors that would predict moderate CKD patients. Thus, randomized controlled trials are essential to deduce the optimal BMI and WC in the case of eGFR loss interventions and the optimal duration for which such interventions should be maintained to achieve satisfactory clinical outcomes.

## Author contributions

**Data curation:** Yaping Zhan, Jiayue Lu.

**Formal analysis:** Minxia Zhu.

**Funding acquisition:** Shang Liu, Weiming Zhang.

**Investigation:** Jiuhong Mei, Pu Zhang, Ronghui Liu.

**Methodology:** Gaojie Xu.

**Resources:** Weiming Zhang, Gaojie Xu.

**Supervision:** Weiming Zhang, Gaojie Xu.

**Validation:** Weiming Zhang, Gaojie Xu.

**Visualization:** Zhaohui Ni, Weiming Zhang, Gaojie Xu.

**Writing – original draft:** Hong Cai.

**Writing – review & editing:** Hong Cai.

## References

[1] Jha V Garcia-Garcia G Lseki K. Chronic Kidney disease: global dimension and perspectives. Lancet 2013;382:260–72. 23727169 10.1016/S0140-6736(13)60687-X

[2] Lozano R Naghavi M Foreman K. Global and regional mortality from 235 causes of death for 20 age groups in 1990 and 2010: a systematic analysis for the Global Burden of Disease Study 2010. Lancet 2013;380:2095–128. 10.1016/S0140-6736(12)61728-0 PMC10790329 23245604

[3] Lakkis JI Weir MR. Obesity and kidney disease. Prog Cardiovasc Dis 2018;61:157–67. 29981350 10.1016/j.pcad.2018.07.005

[4] GBD obesity collaborators. Health effects of overweight and obesity in 195 countries over 25years. N Engl J Med 2017;377:13–27. 28604169 10.1056/NEJMoa1614362 PMC5477817

[5] Aunne D Sen A Prasad M. BMI and all cause mortality: systematic review and non-liner dose-response meta-analysis of 230 cohort studies with 3.74million deaths among 30.3million participants. BMJ 2016;353:i2156. 27146380 10.1136/bmj.i2156 PMC4856854

[6] Berrington de Gonzalez A Hartge P Cerhan JR. Body-mass index and mortality among 1. 46million white adults. N Engl Med 2010;363:2211–9. 10.1056/NEJMoa1000367 PMC3066051 21121834

[7] Global BMI Mortality Collaboration. Body-mass index and all-cause mortality: individual-participant-data meta-analysis of 239 prospective studies in four continents. Lancet 2016. 10.1016/S0140-6736(16)30175-1 PMC4995441 27423262

[8] Bhaskaran K Dos-santos-silva L Leon DA. Association of BMI with overall and cause-specific mortality: a population-based cohort study of 3.6 million adults in the UK. Lancet Diabetes Endocrinol 2018;6:944–53. 30389323 10.1016/S2213-8587(18)30288-2 PMC6249991

[9] Lu JL Molnar MZ Naseer A. Association of age and BMI with kidney function and mortality: a cohort study. Lancet Diabetes Endocrinol 2015;3:704–14. 26235959 10.1016/S2213-8587(15)00128-X PMC4547884

[10] Essam FE Mark JS Hocine T. Waist to hip ratio, body mass index and subsequent kidney disease and death. Am J Kidney Dis 2008;52:29–38. 18511168 10.1053/j.ajkd.2008.02.363 PMC4052757

[11] Postorino M Marino C Tripepi G. CREDIT working group: abdominal obesity and all-cause and cardiovascular mortality in end-stage renal disease. J Am Coll Cardiol 2009;58:1265–72. 10.1016/j.jacc.2008.12.040 19358939

[12] 2018;Bhaskaran K Dos SS Leon DA. Association of BMI with overall and cause-specific mortality: a population-based cohort study of 36 million adults in the UK. 6:944–53. 10.1016/S2213-8587(18)30288-2 PMC6249991 30389323

[13] Levey AS Stevens LA Schmid CH. A New equation to estimate glomerular filtrateion rate. Ann Intern Med 2009;150:604–12. 19414839 10.7326/0003-4819-150-9-200905050-00006 PMC2763564

[14] Elsayed EF Tighiouart H Weiner DE. Waist-to-hip ratio and body mass index as risk factors for cardiovascular events in CKD. Am J Kidney Dis 2008;52:49–57. 18514990 10.1053/j.ajkd.2008.04.002 PMC2693892

[15] Madero M. Body mass index and mortality in CKD. Am J kidney Dis 2008;50:404–11. 10.1053/j.ajkd.2007.06.004 17720519

[16] Janssen I Katzmarzyk PT Ross R. Body mass index, waist circumference, and health risk: evidence in support of current National institutes of Health guidelines. Arch Intern Med 2002;162:2074–9. 14. 12374515 10.1001/archinte.162.18.2074

[17] Sanches FM Avesani CM Kamimura MA. Waist circumference and visceral fat in CKD: a cross-sectional study. Am J Kidney Dis 2008;52:66–73. 18440683 10.1053/j.ajkd.2008.02.004

[18] Yang S Li M Chen Y. Comparison of the correlates between body mass index, waist circumference, waist-to-height ratio, and chronic kidney disease in a rural Chinese adult population. J Ren Nutr 2018;S1051–2276. [Epub ahead of print]. 10.1053/j.jrn.2018.10.008 30581064

[19] Xie L Wang B jiang C. BMI is associated with the development of chronic kidney in hypertensive patients with normal renal function. J Hypertens 2018;36:2085–91. 29957719 10.1097/HJH.0000000000001817

[20] Lu JL Molnar MZ Naseer A. Age and associateon of body mass index with loss of kidney function and mortality. Lancet Diabetes Endocrinol 2015;3:704–14. 26235959 10.1016/S2213-8587(15)00128-X PMC4547884

[21] Foster MC Hwang SJ Porter SA. Fatty kidney, hypertension, and chronic kidney disease: the Framingham Heart Study. Hypertension 2011;58:784–90. 21931075 10.1161/HYPERTENSIONAHA.111.175315 PMC3204377

[22] Tsuboi N Utsunomiya Y Kanzaki G. Low glomerular density with glomerulomegaly in obesity-related glomerulopathy. Clin J Am Soc Nephrol 2012;7:735–41. 22403274 10.2215/CJN.07270711

[23] Knight SF Quigley JE Yuan J. Endothelial dysfunction and the development of renal injury in spontaneously hypertensive rats fed a high-fat diet. Hypertension 2008;51:352–9. 18158349 10.1161/HYPERTENSIONAHA.107.099499 PMC2491336

[24] Okada R Yasuda Y Tsushita K. Glomerular hyperfiltration in prediabetes and prehepertension. Nephrol Dial Transplant 2012;27:1821–5. 22140135 10.1093/ndt/gfr651

[25] Ruggenenti P Porrini EL Gaspari F. Glomerular hyperfiltration and renal disease progression in type 2 diabetes. Diabetes Care 2012;35:2061–8. 22773704 10.2337/dc11-2189 PMC3447826

